# Downregulation of miR-518a-3p activates the NIK-dependent NF-κB pathway in colorectal cancer

**DOI:** 10.3892/ijmm.2015.2145

**Published:** 2015-03-18

**Authors:** L.L. QU, L. HE, X. ZHAO, W. XU

**Affiliations:** 1 Department of Laboratory Medicine, The First Hospital of Jilin University, Changchun, Jilin 130021, P.R. China; 2 Department of Gastroenterology Surgery, The First Hospital of Jilin University, Changchun, Jilin 130021, P.R. China; 3 Department of Hepatology, The First Hospital of Jilin University, Changchun, Jilin 130021, P.R. China

**Keywords:** miR-518a-3p, colorectal cancer, nuclear factor-κB-inducing kinase, nuclear factor-κB activation

## Abstract

The aim of the present study was to investigate the biological role and underlying mechanisms of action of miR-518a-3p in the progression and invasion of colorectal cancer (CRC). Reverse transcription-quantitative PCR (RT-qPCR) was used to examine the mRNA expression levels of miR-518a-3p in 5 CRC cell lines (SW480, SW620, HCT116, HT29 and LoVo) in a normal colonic cell line, NCM460, as well as in tumor tissues with or without metastases. The biological effects of miR-518a-3p were assessed in the CRC cell lines by 3-(4,5-dimethylthiazol-2-yl)-2,5-diphenyltetrazolium bromide (MTT) assay and flow cytometric analysis, and RT-qPCR and western blot analyses were employed to evaluate the expression of miR-518a-3p targets. The regulation of NF-κB-inducing kinase (NIK) by miR-518a-3p was confirmed using luciferase activity assays. Our results revealed that miR-518a-3p was significantly downregulated in the CRC cell lines compared with the normal colonic cell line (P<0.05), as well as in the CRC tissues with distant metastases compared with the tissues without metastases. The downregulation of miR-518a-3p was associated with tumor size, distant metastasis and TNM stage in the patients with CRC. Moreover, the ectopic expression of miR-518a-3p and the inhibition of NIK by RNA interference markedly reduced cell proliferation and enhanced the apoptosis of CRC cells. Further experiments revealed that NIK, a regulator of NF-κB, was a downstream target of miR-518a-3p. The presents findings indicate that miR-518a-3p plays an important role in the progression of CRC by targeting NIK.

## Introduction

In Western countries, colorectal cancer (CRC) is the second leading cause of cancer-related mortality (1). Recent advancements in the therapeutic strategies for this disease have successfully saved the lives of a number of patients with early-stage disease. However, the mortality rate of patients with advanced stages of the disease remains high. Thus, further investigations are urgently required to elucidate the specific molecular mechanisms responsible for the progression of CRC.

In the majority of tumor cells, constitutive activation of nuclear factor-κB (NF-κB) is often observed (2). The abnormal activation of NF-κB contributes to significant cell proliferation and migration in CRC and in other types of cancer (3), and the inhibition of NF-κB activity has been shown to significantly reduce cancer cell growth and enhance cell apoptosis, indicating that NF-κB members are potential therapeutic targets in tumors (4). Mammalian NF-κB members mainly include p65 (RelA), p105/p50, RelB, p100/p52 and c-Rel (5), and two distinct pathways are involved in the regulation of NF-κB activation: the canonical and the non-canonical pathways. The canonical pathway of NF-κB is controlled by the IκB kinase (IKK) complex, which is comprised of IKKα, IKKβ and IKKγ. Through the phosphorylation of inhibitors of κB (IκBs), the IKK complex usually sequesters NF-κB members into the cytoplasm, thereby inhibiting the nuclear translocation of these transcription factors. The non-canonical pathway of NF-κB is regulated by IKKα and the NF-κB-inducing kinase (NIK). NIK, also known as mitogen-activated protein kinase kinase kinase 14 (MAP3K14), activates NF-κB upon the stimulation of tumor necrosis factor (TNF), leading to the activation of the canonical pathway (6). NIK plays a key role in receptor-initiating signaling in the non-canonical (alternative) NF-κB pathway. It has been suggested that the activation of NF-κB by NIK significantly promotes epithelial cell proliferation, the inflammatory response and oncogenic signaling (7). In normal cells, the protein level of NIK is maintained at a normal level by proteasomal degradation; however, aberrant NIK accumulation has been observed in some cancer cells (8). However, studies on NIK accumulation in CRC are limited.

MicroRNAs (miRNAs or miRS) are small non-coding RNAs that are 18–25 nt in length. Through interactions with 3′ untranslated regions (3′ UTRs), miRNAs negatively regulate the expression of a series of target genes. Over the years, a number of studies have demonstrated the important role of miRNAs in cancer pathology, indicating that miRNAs can act as either oncogenes or tumor suppressors (9–11). In many types of tumors, the downregulation of miRNAs and the upregulation of oncogenes have been reported, as was demonstrated for cervical cancer (12). For instance, in high-grade serous ovarian carcinomas, miR-106 has been reported to be significantly upregulated and to enhance cell proliferation and differentiation (13). Furthermore, miR-519 was found to target HuR, thereby reducing cell proliferation and cell cycle progression in various types of tumor (14). For instance, miR-150 and miR-630 induce pancreatic cancer cell apoptosis by targeting insulin-like growth factor 1 (IGF-1R), while miR-21 functions as an anti-apoptotic regulator by targeting pro-apoptotic genes, such as Fas ligand (FasL), phosphatase and tensin homolog (PTEN) and programmed cell death protein 4 (PDCD4) (15). Taken together, these data suggest that miR-518a-3p regulates cell proliferation and cell cycle progression and may be associated with the progression of human cancer.

In this study, we identified a novel miRNA, miR-518a-3p, which regulates the protein level of NIK. Furthermore, our results indicate that the downregulation of miR-518a-3p abnormally activates NF-κB signaling in CRC, thereby defining the significance of miR-518a-3p in the progression of CRC.

## Materials and methods

### Statement

All the subjects who participated in this study provided written informed consent, and this study was approved by the Ethics Committee of Jilin University (Changchun, China).

### Human tissue specimens and cell lines

CRC tissues with distant metastases (n=42) and without distant metastases (n=40) were obtained from 82 patients with CRC who underwent an initial surgery at the First Hospital of Jilin University between June 2009 and January 2011. All the patients had a histological diagnosis of CRC. Following resection, the specimens were snap-frozen in liquid nitrogen and stored at −80°C until RNA extraction.

The human HT-29, HCT116, SW480, SW620 and LoVo CRC cell lines, the 293T embryonic kidney cell line, and the NCM460 normal colonic epithelial cell line were purchased from the American Type Culture Collection (ATCC, Manassas, VA, USA). The cells were cultured in RPMI-1640 medium containing 10% fetal bovine serum in a humidified 37°C incubator supplemented with 5% CO_2_.

### RNA isolation and reverse transcription-quantitative PCR (RT-qPCR)

Total RNA was extracted from the tissues and cells using TRIzol reagent (Invitrogen Life Technologies, Carlsbad, CA, USA). RNA quality and concentration were determined using the NanoDrop 2000 system (Thermo Fisher Scientific, Inc., Wilmington, MA, USA). To quantify miR-518a-3p expression, a TaqMan MicroRNA Assay kit (Applied Biosystems, Foster City, CA, USA) was used, and U6 snRNA was used as a reference. To quantify the NIK mRNA level, a SYBR Premix Ex Taq™ kit [Takara Biotechnology (Dalian) Co., Ltd., Liaoning, Japan] was used, and β-actin expression was used as an endogenous control. Quantitative PCR was performed using the Applied Biosystems 7900 Fast Real-Time PCR system (Applied Biosystems). The data were analyzed using the 2^−ΔΔct^ method.

### Western blot analysis

Western blot analysis was performed as previously described (16). Briefly, total cellular protein was isolated, and the protein concentration was determined using the Bradford DC protein assay (Bio-Rad, Hercules, CA, USA). Forty micrograms of protein were separated by SDS-PAGE and transferred onto polyvinylidene fluoride (PVDF) membranes. The membranes were then incubated with the following primary antibodies: NIK (#4994, 1:1,000), X-linked inhibitor of apoptosis (XIAP; #14334, 1:1,000), FLICE-like inhibitory protein (FLIP; #8510, 1:1,000), B-cell lymphoma-extra large (Bcl-xL; #2764, 1:1,000) (all from Cell Signaling Technology, Inc., Boston, MA, USA) and GAPDH (1:2,000; Santa Cruz Biotechnology, Inc., Santa Cruz, CA, USA), and the proteins were visualized by the ECL procedure (Amersham Biosciences Corp., Piscataway, NJ, USA).

### Oligonucleotide transfection

The hsa-miR-518a-3p mimic, negative control (NC) oligonucleotides, has-miR-518a-3p inhibitor and the scramble oligonucleotides were purchased from Guangzhou RiboBio Co., Ltd. (Guangzhou, China). The cells were plated in a 6-well plate the day prior to transfection. Using Lipofectamine 2000 (Invitrogen Life Technologies), the LoVo cells were transfected with the hsa-miR-518a-3p mimic or NC (50 nmol/l), and the LoVo cells were infected with the has-miR-518a-3p inhibitor or scramble oligonucleotides (100 nmol/l). Twenty-four hours later, the cells were collected, and *in vitro* assays were performed.

### 3-(4,5-Dimethylthiazol-2-yl)-2,5-diphenyltetrazolium bromide (MTT) assay

To examine the effect of miR-518a-3p on cell viability, 5,000 cells/well in 100 *μ*l of medium were seeded in 96-well plates and transfected with miR-518a-3p mimics (50 nM) or negative control (50 nM), as described above. At 24 h after transfection, 20 *μ*l of MTT reagent (Beijing Solarbio Science & Technology Co., Ltd., Beijing, China) was added to the wells and then incubated with the cells for 4 h. After removing the medium, the blue formazan was dissolved in 200 *μ*l of dimethyl sulfoxide (DMSO) (Beijing Solarbio, Scienc e & Technology Co., Ltd.), and the absorbance was measured at 550 nm. Wells containing only LoVo cells served as the blanks.

### Immunofluorescence

The LoVo cells were cultured on 6-well chamber slides and fixed with 4% paraformaldehyde for 10 min at −20°C. The slides were washed in PBS 3 times, and incubated with a polyclonal antibody against NIK (1:50 diluted in PBS with 1% BSA, 50 *μ*l/slide) for 2 h at room temperature. After washing with PBS 3 times (5 min/time), the slides were incubated with TRITC-conjugated anti-mouse IgG (1:100 diluted in PBS with 1% BSA, 50 *μ*l/slide) for 1 h at room temperature. After washing the slides in PBS 3 times, the slides were incubated with Hoechst 33258 (10 *μ*g/ml) for 5 min. The slides were then washed again and examined under a fluorescence microscope (Axio Observer; Zeiss, Göttingen, Germany).

### Hoechst 33258 staining

The LoVo cells were cultured in 6-well plates. After 48 h of transfection with miR-518a-3p mimics, inhibitors or negative control, the cells were washed with PBS and then stained with Hoechst 33258 (10 *μ*g/ml) (Beijing Solarbio Science & Technology Co., Ltd.) for 5 min before being washed 3 times with PBS.

### Quantification of apoptotic cells

To quantify the apoptotic cells, flow cytometry was performed using an Annexin V-fluorescein-5-isothiocyanate Apoptosis Detection kit (BioVision, Inc., Milpitas, CA, USA). Forty-eight hours after transfection with miR-518a-3p mimics (50 nM) or negative control (50 nM), the LoVo cells were placed in a 5-ml tube. The cells were then washed with cold PBS and resuspended at a final concentration of 1×10^6^ cells/ml. FITC-Annexin V (5 *μ*l) and propidium iodide were gently mixed and incubated with the cells for 15 min at room temperature. Within 1 h after incubation, the samples were analyzed by flow cytometry.

### Vector construction and dual-luciferase reporter assays

Before the luciferase assays were performed, the potential miR-518a-3p binding site in the NIK 3′ UTR was predicted using TargetScan (www.targetscan.org) and miRanda (www.microRNA.org). The 3′ UTR of the NIK mRNA and a mutant NIK mRNA were then synthesized and cloned into the *Xba*I site of a pGL3 basic vector (Promega Corp., Madison, WI, USA) downstream of the luciferase stop codon, and these plasmids were designated as pGL3-wt-NIK and pGL3-mt-NIK, respectively. Subsequently, the 293T cells (1×10^5^ cells/well) were cultured in 24-well plates and co-transfected with the pGL3-control (0.4 mg), pGL3-wt-NIK (0.4 mg) or the pGL3-mt-NIK (0.4 mg) plasmid, as well as the pRL-TK luciferase reporters (25 ng/well) and pcDNA-miR-518a-3p (20 nmol/l) or pcDNA-miR-NC (20 nmol/l) using Lipofectamine 2000 (Invitrogen Life Technologies). Forty-eight hours later, the cells were harvested, and the luciferase activity was measured using a Dual-Luciferase Reporter Assay kit (Promega Corp.).

### Inhibition of NIK by RNA interference

NIK-specific shRNA (shNIK) and negative control were purchased from Shanghai GenePharma Co., Ltd. (Shanghai, China) Cells (1×10^5^/well) in a 6-well plate were transfected with 50 nM shNIK or negative control for 48 h using HiPerFect Transfection Reagent (Qiagen GmbH, Düsseldorf, Germany) as described above.

### Statistical analysis

The data are expressed as the means ± SD, and statistical significance was analyzed using the Student’s t-test (two-tailed). All statistical analyses were performed using SPSS 13.0 software or the GraphPad Prism 5.0 software package. The Kaplan-Meier method and the log-rank test were performed to analyze the prognostic significance. A value of P<0.05 was considered to indicate a statistically significant difference in all tests.

## Results

### miR-518a-3p is significantly downregulated in CRC cell lines and CRC tissues with metastases

**Figure 1:**
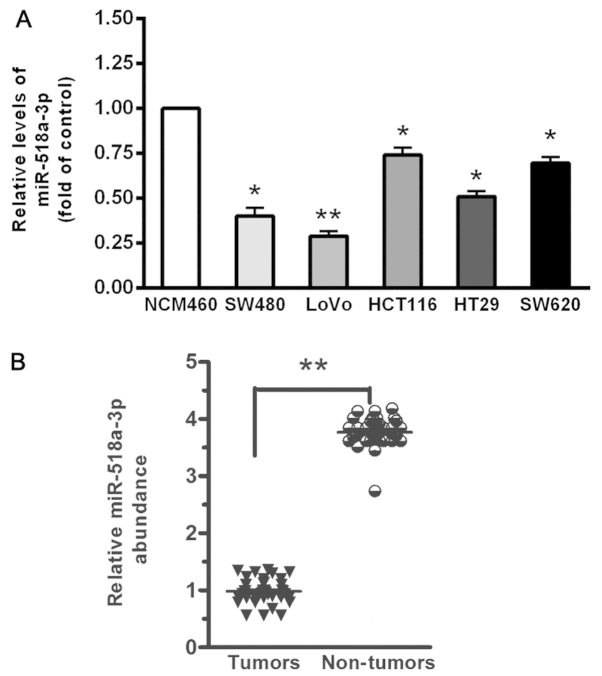
miR-518a-3p is significantly downregulated in colorectal cancer (CRC) cell lines and tissues with distant metastases. (A) Relative expression level of miR-518a-3p in CRC cell lines and in the normal colonic cell line, NCM460 (^*^P<0.05). (B) The relative expression level of miR-518a-3p in CRC tissues with distant metastases (n=42) and without metastases (n=40) (^**^P<0.01).

First, we analyzed the expression of miR-518a-3p in 5 CRC cell lines and in the normal colonic cell line, NCM460. miR-518a-3p was significantly downregulated in the CRC cell lines compared with the NCM460 cells (all P<0.05) (Fig. 1A). Among the 5 CRC cell lines, the LoVo cells showed the lowest miR-518a-3p expression level, and the HCT116 cells exhibited the highest miR-518a-3p level (Fig. 1A). To examine the expression of miR-518a-3p in CRC tissues, the miR-518a-3p level was measured in CRC tissues with metastases (n=42) and normal tissues without metastases (n=40). Of note, miR-518a-3p expression was markedly lower in the CRC tissues than in the normal tissues (P<0.001; Fig. 1B).

### NIK is a direct target of miR-518a-3p

**Figure 2:**
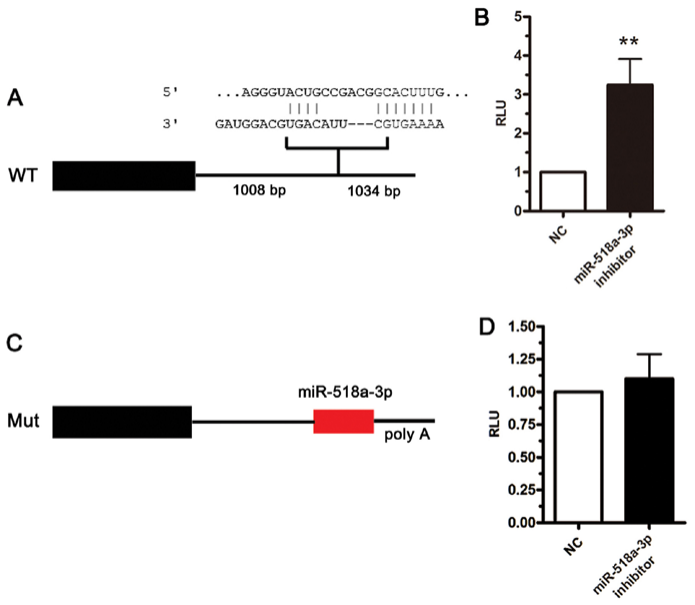
Nuclear factor (NF)-κB-inducing kinase (NIK) is a direct target of miR-518a-3p. (A) Schematic of the miR-518a-3p target sites in the NIK 3′ untranslated regions (3' UTRs). (B) Reporter-based miR-518a-3p target gene screening. A series of 3′ UTR-luciferase reporters was transfected into SW620 cells with or without miR-518a-3p-specific inhibitory RNA (anti-miR-518a-3p) or negative control RNA (NC). The relative values of the dual-luciferase assays are presented, and the data are presented as the means ± SD of 3 independent experiments. (C) Mutation-induced reporters. The red box represents the mutated target region. (D) miR-518a-3p negatively regulates the NIK 3' UTR, as analyzed by reporter assays (n=4, means ± SD). The data represent the means ± SEM, n=3 independent experiments. ^**^P<0.01 vs. control.

To determine the functional significance of the downregulation of miR-518a-3p, we attempted to identify the target genes of miR-518a-3p using computational algorithms. A computational search predicted one binding site for miR-518a-3p in the NIK 3′ UTR (Fig. 2A). To experimentally identify the target genes of miR-518a-3p, we performed reporter-based screens as described below. Luciferase-3' UTR reporter assays demonstrated a marked negative effect against upstream gene expression by the NIK 3′ UTR sequence. As shown in Fig. 2B, treatment with a miR-518a-3p inhibitor increased NIK 3′ UTR reporter activity, suggesting the involvement of endogenous miR-518a-3p in NIK down-regulation. To identify the regulatory sequence in the 3' UTR of NIK, we constructed additional reporters with mutated sequences in the seed region (Fig. 2C). The reporter containing the mutated miR-518a-3p seed sequence prevented the effects of anti-miR-518a-3p treatment (Fig. 2D), and miR-518a-3p inhibition inversely rescued the NIK level, which revealed that the cellular miR-518a-3p level negatively affected that of the NIK protein through its 3' UTR sequence. These lines of evidence collectively demonstrate that miR-518a-3p recognizes and regulates NIK mRNA through specific binding to its 3′ UTR.

### miR-518a-3p negatively regulates NF-κB signaling through NIK expression

**Figure 3:**
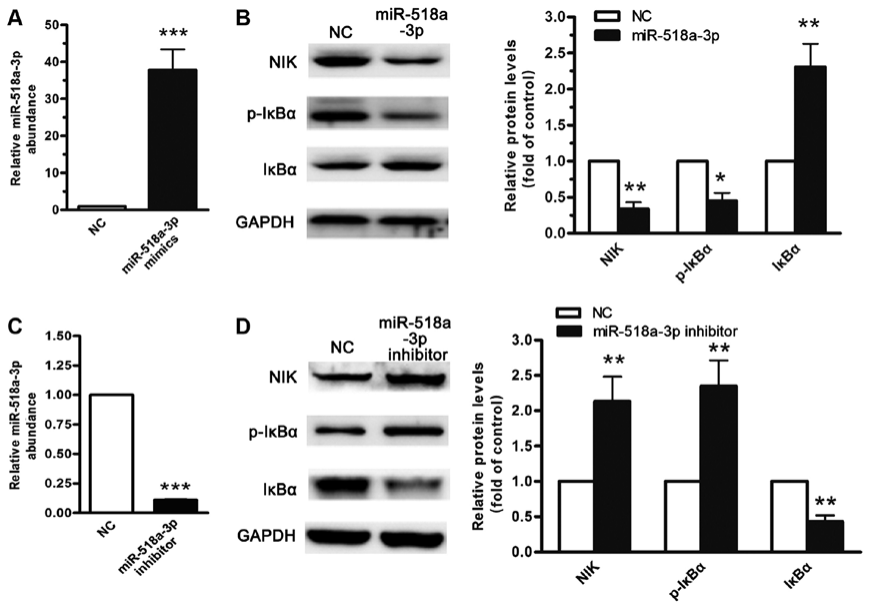
miR-518a-3p negatively regulates the nuclear factor (NF)-κB pathway by inhibiting NF-κB-inducing kinase (NIK) expression. NIK protein (right panel of B and D) levels in LoVo cells, as measured by western blot analysis. Treatment with miR-518a-3p (A and B) mimics or (C and D) inhibitor resulted in the suppression or accumulation of NIK expression, respectively. The data represent the means ± SEM, n=3 independent experiments. ^*^P<0.05, ^**^P<0.01 vs. control.

The transient introduction of the miR-518a-3p precursor into the LoVo cells resulted in the downregulation of NIK at the protein level and was associated with the downregulation of the phosphorylated IκBα level and NF-κB activity (Fig. 3A and B). By contrast, the inhibition of miR-518a-3p resulted in the accumulation of NIK protein expression in the LoVo cells (Fig. 3C and D). The manipulation of the miR-518a-3p level clearly indicated that the miR-518a-3p level negatively correlated with cellular NF-κB activity. These results collectively demonstrate that miR-518a-3p inhibits the basal and receptor-initiated activities of the non-canonical NF-κB pathway and that miR-518a-3p plays a critical role in the negative regulation of the NF-κB pathway by manipulating the expression of NIK.

### miR-518a-3p suppresses LoVo cell growth by inhibiting NF-κB

**Figure 4:**
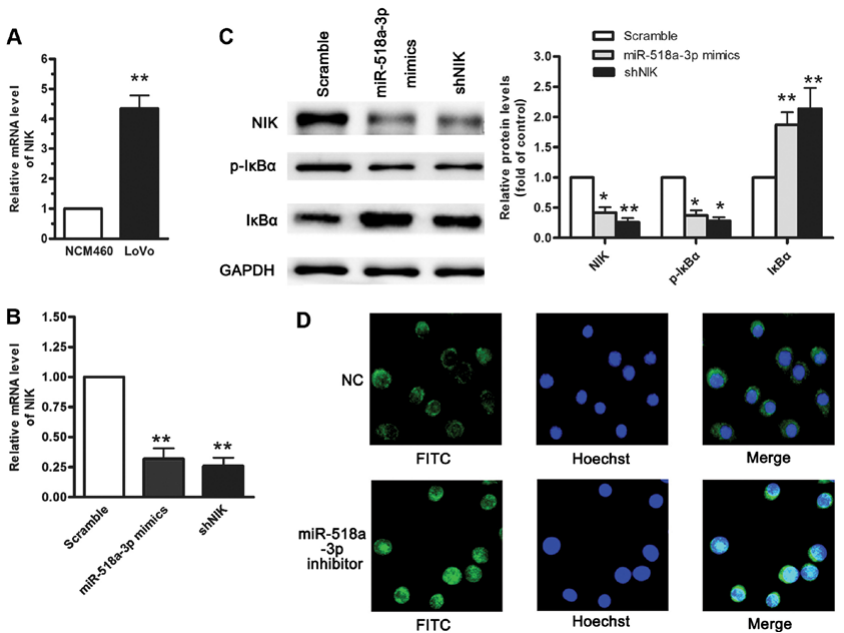
The loss of miR-518a-3p is responsible for constitutive nuclear factor (NF)-κB activation, abnormal cell growth and resistance to apoptosis in NCM460 cells. (A) The mRNA level of NIK was detected using RT-PCR in LoVo and NCM460 cells. (B) miR-518a-3p restoration by a retroviral vector inhibits NIK RNA accumulation in NCM460 cells. The quantification of NIK and mature miR-518a-3p expression is shown (n=3, means ± SD). (C) miR-518a-3p or NIK-specific shRNA (shNIK) expression downregulates NIK protein expression and inhibits the downstream non-canonical NF-κB pathway in NCM460 cells. (D) Increased nuclear translocation of RelA due to the expression of miR-518a-3p inhibitor in NCM460 cells. The data represent the means ± SEM, n=3 independent experiments. ^*^P<0.05, ^**^P<0.01 vs. control.

Although it has been documented that the abnormal accumulation of NIK in cells acts as a constitutive activator of the NF-κB pathway (17), the mechanisms underlying the overproduction of NIK remain to be elucidated. RT-qPCR revealed that the abnormal accumulation of NIK in the LoVo cells acts as a constitutive activator of the NF-κB pathway when compared with the NCM460 cells (Fig. 4A). To investigate the functional roles of NIK and miR-518a-3p, we established NCM460 cells stably expressing miR-518a-3p or NIK-specific shRNA (shNIK) using retroviral vectors. RT-qPCR and western blot analysis revealed that the enforced expression of miR-518a-3p or transfection with shNIK reduced the mRNA and protein levels of NIK, as well as the levels of p-IκBα, but not those of IκBα (Fig. 4B and C). Furthermore, the enhanced translocation of nuclear RelA increased the activity of the canonical and non-canonical NF-κB pathways (Fig. 4D). The re-expression of NIK led to the activation of NF-κB that was inhibited by miR-518a-3p, suggesting a reciprocal association between the level of miR-518a-3p and that of NIK.

### miR-518a-3p promotes apoptosis by inhibiting NF-κB

**Figure 5:**
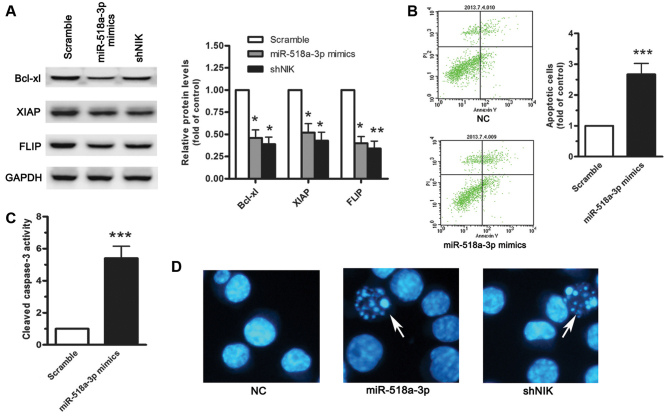
(A) The suppression of NIK by miR-518a-3p or shNIK resulted in the downregulation of a subset of genes involved in resistance to apoptosis, including Bcl-xL, XIAP and FLIP. (B) Transfection with miR-518a-3p mimics promoted the apoptosis of the LoVo cells. (C) Overexpression of miR-518a-3p led to the activation of caspase-3. (D) Hoechst staining was applied to detect cell apoptosis when LoVo cells were transfected with miR-518a-3p or shNIK. The data represent the means ± SEM, n=3 independent experiments. ^*^P<0.05, ^**^P<0.01, ^***^P<0.001 vs. control.

We hypothesized that the miR-518a-3p-mediated NF-κB modulation may affect cellular apoptosis as numerous studies have demonstrated that NF-κB activation is a strong anti-apoptotic factor in CRC cells (6,7). We found that the suppression of NIK by miR-518a-3p or shNIK resulted in the downregulation of a subset of genes involved in resistance to apoptosis, including Bcl-xL, XIAP and FLIP (Fig. 5A), which suggests that miR-518a-3p plays a role in promoting apoptosis through the inhibition of NF-κB activity. To assess the biological function of miR-518a-3p in apoptotic signals, we transfected miR-518a-3p mimics into the LoVo cells, and found that trans-fection with miR-518a-3p mimics promoted the apoptosis of the LoVo cells (Fig. 5B). In addition, miR-518a-3p overexpression led to the activation of caspase-3 (Fig. 5C). Collectively, these findings indicate that miR-518a-3p mediates apoptosis through the suppression of NIK in colonic cell lines. To demonstrate the role of miR-518a-3p in cancer cell survival, we examined whether the transfection of miR-518a-3p mimics resulted in a killing effect against the cancer cells, and the number of apoptotic cells was determined by Hoechst staining. The results revealed that the expression of miR-518a-3p facilitated tumor cell death (Fig. 5D). Since the suppression of NIK by shRNA also had a strong killing effect, NIK and NF-κB may thus be crucial players in the survival of CRC cells. Taken together, these lines of experimental evidence definitively support two notions: i) miR-518a-3p functions as a tumor suppressor in CRC cells; and ii) NIK-regulated NF-κB is important for CRC cell survival.

## Discussion

In various types of tumor, including Hodgkin's lymphoma, breast cancer, prostate cancer and CRC, the constitutive activation of NF-κB significantly promotes abnormal cancer cell proliferation and inhibits cell death (18-20). Furthermore, NF-κB plays key roles in different cellular functions, such as inflammation, innate immunity and lymphocytic development (21). A deeper understanding of NF-κB signaling may thus lead to progress being made in the determination of the molecular pathology of various types of cancer, such as CRC.

Increasing evidence has suggested the important role of miRNAs in cancer progression (22,23). In CRC, some miRNAs have been suggested to widely participate in the pathology of tumorigenesis and metastasis. For instance, miR-21, miR-31 and miR-192 have been shown to increase the resistance of CRC cells to 5-fluorouracil (5-FU) (24–26), and a polymorphism in pre-miR-27a has been shown to significantly correlate with the risk of developing CRC (27). Furthermore, miR-182 is increased in colorectal carcinoma, suggesting that it is a potential prognostic factor for CRC (28–30). However, the role of miR-518a-3p in CRC remaines to be elucidated. In this study, we mainly focused on the functional significance of miR-518a-3p in abnormal CRC cell proliferation. Our results revealed a profound downregulation of miR-518a-3p in all CRC cases, suggesting that the loss of miR-518a-3p is a prerequisite for the development of CRC. These results indicate that the downregulation of miR-518a-3p may be a common occurrence in CRC and that miR-518a-3p may be used as a biomarker to predict clinical outcome and metastasis in patients with CRC.

Using miRBase, we first identified NIK as a possible target gene of miR-518a-3p. In the present study, we identified NIK as a target of miR-518a-3p. First, luciferase-3' UTR reporter assays revealed that the NIK 3' UTR sequence plays a role in its negative regulation. By combining a specific inhibitor and mutations in the miR-518a-3p-binding site, we demonstrated that miR-518a-3p recognizes and negatively regulates the 3' UTR of NIK (Fig. 2A). Second, by introducing an miR-518a-3p precursor or inhibitor, we demonstrated that the amount of miR-518a-3p inversely correlates with the levels of expression and downstream signaling of NIK. Collectively, we provide definitive evidence demonstrating that miR-518a-3p negatively regulates NIK expression and activity. It is well known that the NIK level directly activates NF-κB signaling in various cell types (7), and we experimentally demonstrated that the negative role of miR-518a-3p in cytokine-induced NIK accumulation is widely important in the non-**canonical regulation** of NF-κB in CRC cell types.

Furthermore, the overexpression of miR-518a-3p and inhibition by shNIK significantly inhibited CRC cell proliferation and enhanced CRC cell apoptosis. Moreover, the restoration of miR-518a-3p suppressed NF-κB activity in the CRC cells and led to the impairment of the proliferative capacity and enhanced the apoptosis of the cells. Our results indicate that the suppression of NF-κB enhances CRC cell death, which is in line with previous observations (18). Since it is highly possible that miR-518a-3p and relevant factors are vital for NF-κB signaling, their aberrant expression would be of great importance to the abnormal signaling and clinical outcomes of CRC.

In the present study, we found that the decreased expression of miR-518a-3p and the elevated expression of NIK lead to the activation of NF-κB in CRC cells. We also demonstrated that the restoration of miR-518a-3p partially impaired the NF-κB-induced aberrant CRC cell proliferation. Furthermore, given that NF-κB is a pivotal transcriptional regulator in normal and oncogenic functions, the understanding of the role of epigenetic regulators and miR-518a-3p in NF-κB signaling may enhance our understanding of the molecular mechanisms of CRC cell function. These findings suggest that an aberrant gene expression pattern correlates with the malignant phenotype, which provides important clues as to the clinical manifestations and may help identify therapeutic targets in CRC cells.

In conclusion, we demonstrate that the downregulation of miR-518a-3p is responsible for oncogenic NF-κB activation and for the malignant phenotypes of CRC. Moreover, our results provide evidence indicating that miR-518a-3p is an important tumor suppressor.
